# The role of O-GlcNAcylation in bone metabolic diseases

**DOI:** 10.3389/fphys.2024.1416967

**Published:** 2024-06-10

**Authors:** Yajing Yang, Xuchang Zhou, HuiLi Deng, Li Chen, Xiaolin Zhang, Song Wu, Aiqun Song, Fengxia Liang

**Affiliations:** ^1^ College of Acupuncture-Moxibustion and Orthopedics, Hubei University of Chinese Medicine, Wuhan, China; ^2^ School of Sport Medicine and Rehabilitation, Beijing Sport University, Beijing, China; ^3^ School of Medicine, Xiamen University, Xiamen, China; ^4^ Hubei Shizhen Laboratory, Wuhan, China; ^5^ Hubei Provincial Collaborative Innovation Center of Preventive Treatment by Acupuncture and Moxibustion, Wuhan, China; ^6^ University of Chinese Medicine (Hubei Provincial Hospital of Traditional Chinese Medicine), Wuhan, China

**Keywords:** O-GlcNAcylation, bone, cartilage, osteoporosis, osteoarthritis, osteosarcoma

## Abstract

O-GlcNAcylation, as a post-translational modification, can modulate cellular activities such as kinase activity, transcription-translation, protein degradation, and insulin signaling by affecting the function of the protein substrate, including cellular localization of proteins, protein stability, and protein/protein interactions. Accumulating evidence suggests that dysregulation of O-GlcNAcylation is associated with disease progression such as cancer, neurodegeneration, and diabetes. Recent studies suggest that O-GlcNAcylation is also involved in the regulation of osteoblast, osteoclast and chondrocyte differentiation, which is closely related to the initiation and development of bone metabolic diseases such as osteoporosis, arthritis and osteosarcoma. However, the potential mechanisms by which O-GlcNAcylation regulates bone metabolism are not fully understood. In this paper, the literature related to the regulation of bone metabolism by O-GlcNAcylation was summarized to provide new potential therapeutic strategies for the treatment of orthopedic diseases such as arthritis and osteoporosis.

## 1 Introduction

**FIGURE 1:**
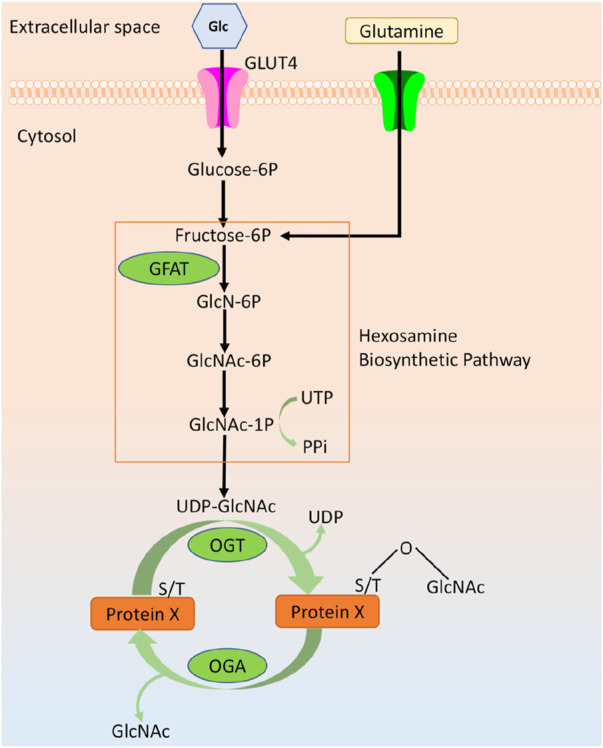
Overview of O-GlcNAcylation.

O-linked β-N-acetylglucosamine (O-GlcNAc) modification is an inducible, reversible, and dynamic post-translational modification of proteins, which is widely found in a variety of multicellular life forms such as plants, animals, and worms and fungi (Shi et al., 2014). It has been reported that the target proteins of O-GlcNAc are mainly cytoplasmic and nuclear proteins, including histones, transcriptases, transcription factors, kinases, structural proteins, and other proteins, with more than tens of thousands of species. O-GlcNAc can participate in a variety of complex processes such as intracellular gene expression, subcellular localization, metabolic immunity, cellular signaling and protein homeostasis through modification of various functional proteins, which play important regulatory roles in the physiological and pathological processes of the organism (Lee B. E. et al., 2021). O-GlcNAc biosynthesis is regulated by two highly conserved enzymes: the O-GlcNAc transferase (OGT) and the O-GlcNAc glycosidase (O-GlcNAcase, OGA), where OGT is mainly localized in the nucleus and OGA is mainly localized in the cytoplasm (Nagel et al., 2013). OGT attaches a single N-acetylglucosamine derived from the UDP-GIcNAc group, the end product of the hexosamine biosynthetic pathway (HBP), to a serine (Ser) or threonine (Thr) residue of the target protein. Conversely, OGA is responsible for removing GlcNAc from the hydroxyl group of the target protein. UDP-GlcNAc synthesized by HBP is influenced by metabolic pathways from glucose, amino acids, fatty acids and nucleic acids. Therefore, O-GlcNAc and HBP are capable of sensing extracellular changes in glucose, fatty acids, and many other factors, including hormonal, immunological and stress cues (Ruan et al., 2013; Hardivillé and Hart, 2014) (Figure 1).

Bone metabolism involves a variety of osteocytes and signaling pathways, of which bone formation dominated by osteoblasts and bone resorption dominated by osteoclasts are two important physiological processes of bone metabolism. The dynamic balance of bone formation and bone resorption is the foundation of bone homeostasis. The osteogenic pattern of bone development includes intramembranous osteogenesis and endochondral osteogenesis. The intramembranous osteogenesis process involves the direct differentiation of mesenchymal stem cells (MSCs) into osteoblasts, which produce osteoids and are encapsulated in the matrix and gradually transformed into osteocytes, while the bone matrix undergoes mineralization. Endochondral osteogenesis is the differentiation of mesenchymal cells into cartilage by aggregation into clusters. Chondrocytes produce bone matrix to form hyaline cartilage, which is followed by differentiation and mineralization of cells in the inner layer of the cartilage endosteum towards osteogenesis and ultimately the formation of cortical bone (Thompson et al., 2015). Abnormalities in osteogenic and chondrogenic differentiation are closely associated with the initiation and progression of bone metabolic diseases such as osteoporosis (OP) and osteoarthritis (OA) (Ahn et al., 2016). Previous studies have shown that high blood glucose levels can regulate the expression and function of osteoblast genes, thereby affecting bone formation and ultimately leading to insulin-dependent diabetic OP. The underlying mechanism may be closely related to the increase of protein O-GlcNAcylation by high blood glucose. Accumulating evidence suggests that aberrant upregulation of O-GlcNAcylation can be involved in the pathogenesis of multiple human diseases such as diabetes, cancer, inflammation and neurodegeneration (Lee B. E. et al., 2021; Ouyang et al., 2022). Importantly, excessive and aberrant protein O-GlcNAcylation affects chondrocyte differentiation and maintenance of bone metabolic homeostasis by regulating multiple signaling pathways (Koyama and Kamemura, 2015; Takeuchi et al., 2016; Nikolaou et al., 2017). Thus, the protein O-GlcNAcylation may be closely associated with the onset and progression of several bone metabolism-related diseases. In this paper, the literature related to O-GlcNAcylation and bone-related diseases was reviewed to reveal the potential regulatory mechanisms of O-GlcNAcylation and bone metabolism, suggesting that targeting and regulating O-GlcNAcylation may be a potential therapeutic strategy for bone metabolic diseases.

## 2 Overview of O-GlcNAcylation

OGT and OGA are currently the only enzymes capable of regulating intracellular O-GlcNAcylation homeostasis in response to a variety of intrinsic and extrinsic stimuli, such as oxidative stress, aging, and altered metabolic status (Yang and Qian, 2017). OGT is mainly expressed in three isoforms: nucleoplasmic OGT (ncOGT), mitochondrial OGT (mOGT), and short OGT (sOGT), which are distinguished in terms of length, subcellular localization, and biological roles. ncOGT, the most classical and well-studied, is involved in biological activities such as gene transcription, proteasomal degradation, and stress tolerance by regulating the vast majority of proteins O-GlcNAcylation in the nucleus and cytoplasm (Chatham et al., 2021). mOGT is predominantly distributed in mitochondria, while sOGT is widely found in cell membranes, nuclei, and mitochondria. mOGT and sOGT perform specific biological functions such as energy production and cell survival (Yang and Qian, 2017). All three OGT isoforms have the same two catalytic domains, as well as a putative phosphatidylinositol (3,4,5)-trisphosphate (PIP3)-binding domain. However, studies of the crystal structure of the human OGT-UDP-peptide complex were unable to confirm the presence of the PIP3 domain (Chatham et al., 2021). OGA is divided into three subtypes: fOGA (full-length O-GlcNAcase), vOGA (variant O-GlcNAcase), and sOGA (shortest O-GlcNAcase). OGA is able to hydrolyze glycosidic bonds after anchoring O-GlcNAc through hydrogen bonds on its active center residues (Comtesse et al., 2001; Gao et al., 2001; Elbatrawy et al., 2020). Previous studies have found that the increase in overall O-GlcNAcylation levels under acute stress is modulated by an increase in OGT and a decrease in OGA. However, in chronic diseases such as cardiac hypertrophy and osteoarthritis, an increase in O-GlcNAc is associated with a concomitant upregulation of OGT and OGA expression (Belke, 2011; Andrés-Bergós et al., 2012; Lunde et al., 2012). Once O-GlcNAcylation homeostasis is disrupted, feedback regulation of both OGT and OGA expression exists to maintain O-GlcNAcylation homeostasis (Andrés-Bergós et al., 2012; Yuzwa et al., 2012; Zhang et al., 2014; Decourcelle et al., 2020). Notably, the regulation of OGA expression occurs at the mRNA level, which may involve epigenetic mechanisms, and the regulation of OGT expression occurs at the protein level, which may involve translational regulation (Lin et al., 2021).

With the development and wide application of mass spectrometry, it has been found that all proteins known to be able to be modified by O-GlcNAc may also undergo other types of post-translational modifications of proteins, such as phosphorylation, ubiquitination, methylation, acetylation, etc., suggesting that these post-translational modification patterns are not isolated, but can interact with each other to jointly regulate the function and activity of proteins. Current research focuses on the mutual regulation of O-GlcNAc modification and phosphorylation modification. The interactions between O-GlcNAcylation and phosphorylation modifications for specific proteins may be antagonistic or synergistic. Four main modes of interaction have been reported for these two post-translational modifications: 1) O-GlcNAc and phosphorylation crosstalk with each other at the same threonine/serine residue sites; 2) crosstalk between O-GlcNAcylation and phosphorylation at neighboring sites; 3) crosstalk occurs at distant sites: despite being far apart in terms of protein sequence, these modifications can still be in close spatial proximity, leading to crosstalk between O-GlcNAcylation and phosphorylation; 4) Mutual influence by regulating the activity or localization of each other’s enzymes (Hu et al., 2010; Zhong et al., 2015). Recent studies have shown that knockdown of PP2A (protein phosphatase 2) to promote phosphorylation in osteoblasts induces translocation of OGT from the nucleus to the cytoplasm and reduces the level of OGT expression, confirming that there is a significant positive correlation between the levels of PP2A and OGT expression, and suggesting that phosphorylation and O-GlcNAcylation compete with each other (Sitosari et al., 2023). In contrast, another study on the effect of O-GlcNAcylation levels on the role of synaptic transmission and plasticity in the mouse hippocampus indicated that an increase in the hippocampal synaptic protein O-GlcNAcylation could concomitantly contribute to the upregulation of phosphorylation modifications (Tallent et al., 2009). Thus, O-GlcNAc modification and phosphorylation modification collaborate and implicate each other to maintain normal cellular life activities.

## 3 O-GlcNAcylation and bone metabolism

### 3.1 O-GlcNAcylation and osteoclasts

Bone resorption is a physiological behavior dominated by osteoclasts and characterized by a decrease in the volume and density of bone tissue, which is controlled by a complex network of molecules, including hormones, chemokines, and cytokines (Cappariello et al., 2014; Ikeda and Takeshita, 2016). Osteoclasts, as the only cells active in bone resorption, are tartrate-resistant acid phosphatase (TRAP)-positive multinucleated cells originating from the hematopoietic monocyte-macrophage system (Cappariello et al., 2014; Ikeda and Takeshita, 2016; Soysa and Alles, 2016). Osteoclast differentiation is a highly dynamic process susceptible to subtle changes in the external environment. Osteoclast differentiation is mainly regulated by macrophage colony-stimulating factor (M-CSF) and receptor activator of nuclear factor-κB ligand (RANKL) (Teitelbaum, 2000; Pettit et al., 2001; Takayanagi et al., 2002). Previous studies have shown that blood glucose is the main energy source for osteoclasts to perform bone resorption functions (Hadjidakis et al., 2006; Catalfamo et al., 2013; An et al., 2019). HBP is a mediator of the interplay between glucose flux, cell signaling, and epigenetic regulation of cellular differentiation (Yuzwa et al., 2012). Extracellular glucose flux can regulate intracellular O-GlcNAcylation levels through the HBP pathway and further affect osteoclastogenesis. Tomoharu et al. (Takeuchi et al., 2016) first demonstrated a negative regulatory relationship between O-GlcNAcylation and osteoclast differentiation. The results showed that GlcNAc (N-acetylglucosamine) treatment led to an increase in the overall level of O-GlcNAcylation in RANKL-induced RAW264 cells, which inhibited RANKL-dependent osteoclastogenic differentiation of murine RAW264 cells. Moreover, OSMI, a specific inhibitor of OGT, significantly inhibited osteoclast differentiation, while exogenous GlcNAc treatment significantly increased osteoclast formation. Specific knockdown of OGT in mouse osteoblasts inhibited osteoclast formation and downregulated key markers associated with osteoclast differentiation and function, thereby alleviating the inflammation-induced reduction in bone loss (Taira et al., 2023). Other studies further elucidated the potential mechanism by which O-GlcNAcylation negatively regulates the process of osteoclast differentiation (Takeuchi et al., 2017; Takeuchi et al., 2020). Takeuchi et al. (2020) used Thiamet G (a specific inhibitor of OGA) to induce an increase in the level of O-GlcNAcylation and found that osteoblasts showed significant inhibition of differentiation in all three different cell types: mouse primary bone marrow cells, RAW264cells, and human peripheral blood mononuclear cells (PBMCs). Further, the knockdown of OGT using siRNA significantly promoted osteoclast differentiation in RAW264 cells. In addition, previous studies have shown that antibodies against citrullinated vimentin are capable of inducing osteoclastogenesis (Harre et al., 2012; Tarbet et al., 2018). Citrullinated vimentin may promote osteoclastogenesis through O-GlcNAcylation (Takeuchi et al., 2020). Thus, the above results suggest that O-GlcNAcylation homeostasis is closely related to the regulation of osteoclast differentiation. Promoting total cellular O-GlcNAcylation levels may be a potential strategy to inhibit osteoclast differentiation.

Notably, Koyama and Kamemura (2015) found that changes in overall levels of O-GlcNAcylation had no effect on osteoclast differentiation of RAW264 cells. This discrepancy may be due to differences in conditions (e.g., cell density, RANKL concentration, and assay conditions) for inducing osteoclast differentiation. Furthermore, to assess osteoclast differentiation, Koyama and Kamemura (2015) detected TRAP mRNA expression as well as TRAP activity, which can be detected in the early stages of osteoclast differentiation, whereas Tomoharu et al. (Takeuchi et al., 2016) assessed the number of osteoclasts and the area of bone resorption, which was mainly used to assess the late stages of osteoclast differentiation. Moreover, a study *in vitro* and *in vivo* experiments showed that increased O-GlcNAcylation plays an important role in promoting osteoclast differentiation (Kim et al., 2021). Recent studies have found that O-GlcNAcylation levels gradually increase during the early stages of osteoclastogenesis but gradually decline to baseline levels during the maturation stage (Li et al., 2022). Increased osteoclast differentiation and activity leads to a shift in bone metabolic homeostasis toward bone resorption in rheumatoid arthritis (RA). Some scholars found that the O-GlcNAcylation levels of immature osteoclasts were all significantly higher than those of mature osteoclasts by comparing the synovial tissues of RA patients and healthy individuals. Further, O-GlcNAcylation levels of immature osteoclasts were found to be significantly higher in the TNF-α overexpressing arthritis transgenic mouse than in the control group (Li et al., 2022). The study further verified that OGT or OGA-related targeted drug inhibition or gene knockout could prevent osteoclast differentiation in the early and late stages of differentiation, respectively (Li et al., 2022). Thus, the osteoclast differentiation may be dynamically regulated by O-GlcNAcylation levels. Furthermore, several pivotal pathways in the regulation of osteoclastogenesis by O-GlcNAcylation have been identified (Li et al., 2022). The initial increase in O-GlcNAc promotes cytokine signaling and metabolic adaptation, thereby facilitating the differentiation of osteoclast precursors, whereas the later downregulation of O-GlcNAc stimulates the rearrangement of the actin cytoskeleton and enhances cell-cell fusion, thereby promoting osteoclast maturation (Li et al., 2022). In addition, Li et al. (2022) found that the effect of O-GlcNAcylation on osteoclast differentiation may be due to the fact that O-GlcNAcylation targets NUP153 and interacts with MYC, which promotes the nuclear translocation of MYC and thus increases the transcriptional activity of MYC by mass spectrometry and transcriptome sequencing analysis (As shown in Figure 1) (Li et al., 2022). NUP153 is a key regulator of RNA splicing (Yoo et al., 2011). MYC is a central component of the network of regulatory genes that drive osteoclast lineage commitment (Caputo et al., 2021), triggering metabolic reprogramming during osteoclast differentiation (Bae et al., 2017).

NF-κB is an important regulatory pathway for RANKL-induced osteoclast differentiation (Boyce, 2013). In the classical NF-κB signaling pathway, NF-κB binds to the NF-κB inhibitor IκBα to form a complex present in the cytoplasm in a resting state. Activation of NF-κB by RANKL and M-CSF triggers activation of the IκBα kinases IKKs, which phosphorylates or ubiquitinates IκBα for degradation and separation from NF-κB. Subsequently, NF-κB translocates to the nucleus and binds to the promoter regions of osteoclast-associated genes, which in turn regulate osteoclast gene transcription. Previous studies have found that several key proteins in the NF-κB pathway, such as p65, c-Rel, IKKα, and IKKβ, can be modified with O-GlcNAc (Li et al., 2019). Some scholars found that upregulation of O-GlcNAcylation can inhibit NF-κB p65 phosphorylation and increase the binding of NF-κB to IκBα, thus preventing NF-κB from translocating into the nucleus to inhibit the expression of downstream transcriptional regulators such as NFATc1 as well as osteoclast specific genes including TRAP, histone K (CtsK), and MMP-9, which ultimately impede the osteoclast differentiation (Zou et al., 2009; Xing et al., 2011; Takeuchi et al., 2016). However, in contrast, another study found that inhibition of O-GlcNAcylation of NF-κB-p65 and NFATc1 with OSMI-1 or knockdown of OGT, respectively, both hindered osteoclast differentiation (Kim et al., 2021). The possible reason for these differences is that different subunits of NF-κB undergo O-GlcNAc modifications that exert different regulatory effects.

**FIGURE 2:**
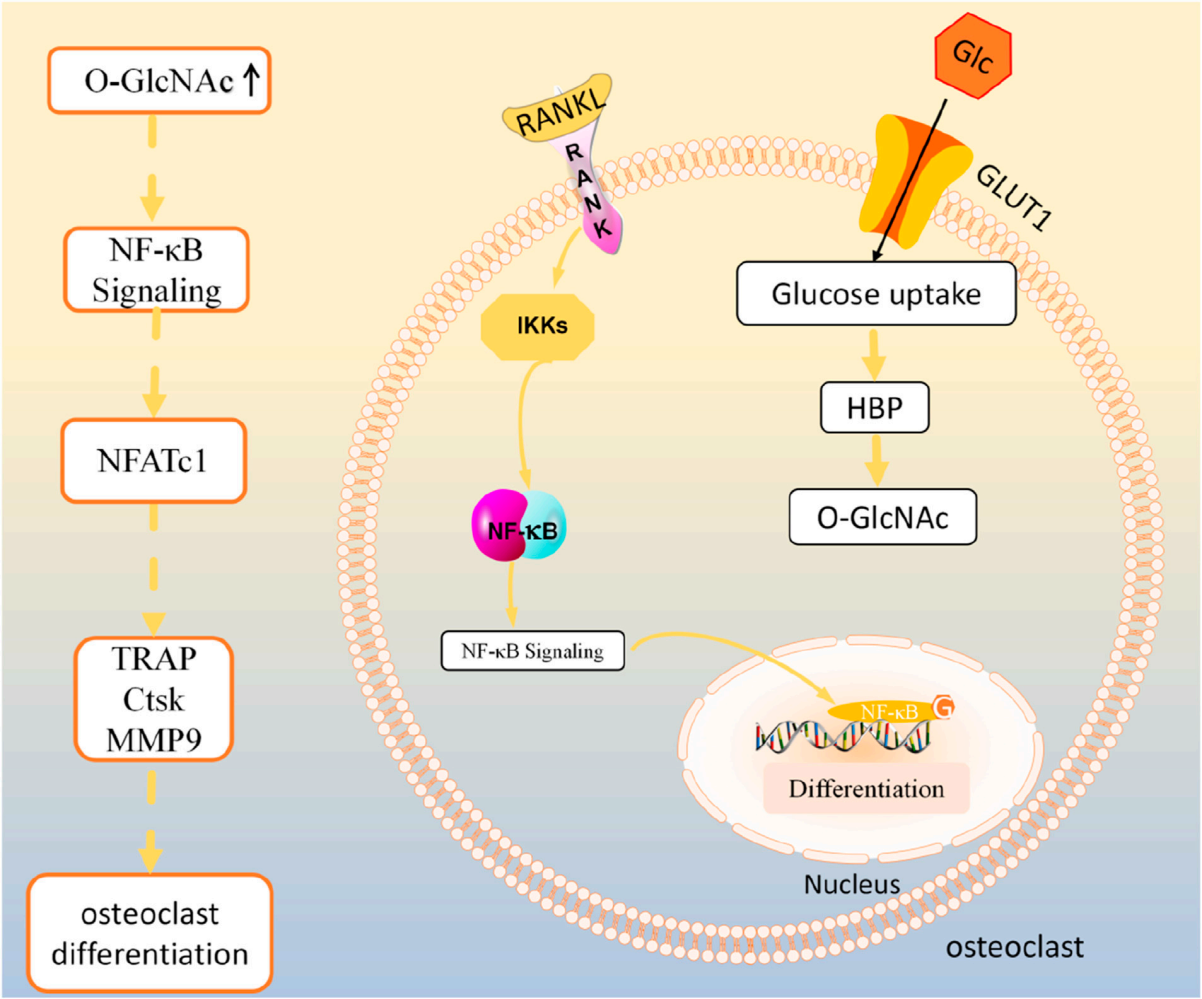
O-GlcNAcylation regulates osteoclast activity.

In summary, O-GlcNAcylation showed different regulatory effects at different stages of osteoclast differentiation (as shown in Figure 2). Targeted regulation of O-GlcNAcylation during osteoclast differentiation may offer a potential clinical therapeutic strategy for the treatment of bone metabolism disorders resulting from abnormal osteoclast differentiation. Undoubtedly, it would be promising for clinical applications to focus on whether changes in O-GlcNAcylation levels play a significant role in the post-translational modification of osteoclast differentiation marker proteins.

### 3.2 O-GlcNAcylation and osteoblasts

Osteoblasts are the main functional cells of bone formation and are responsible for the synthesis, secretion and mineralization of the bone matrix (Chhana and Dalbeth, 2014). Abnormal osteogenic differentiation is one of the most important pathogenic causes of bone metabolism-related diseases, which are manifested by decreased bone volume and sparse trabeculae in bone tissue (Jensen et al., 2021; Zhang et al., 2021). Takahiro et al. (Koyama and Kamemura, 2015) used two O-GlcNAc-specific antibodies for the first time to demonstrate that overall O-GlcNAcylation is elevated in MC3T3-E1 cells during the early stages of osteogenic differentiation. Functional validation by inhibiting the expression of OGT and OGA, respectively, revealed that the O-GlcNAcylation level of the proteins positively regulated the early marker of osteogenic differentiation, ALP, and the late markers, OCN and bone sialoprotein (BSP), as well as the calcification of extracellular matrix (ECM), which promoted osteoblast differentiation (Komori, 2006; Nagel and Ball, 2014; Koyama and Kamemura, 2015; Weng et al., 2021). Thus, Takahiro et al. proposed that the efficiency of osteoblast differentiation could be regulated by controlling the level of overall O-GlcNAcylation. Notably, although the overall level of O-GlcNAcylation was elevated during the early stages of osteogenic differentiation, the overall level of O-GlcNAcylation did not continue to increase after 21 days of osteogenesis-induced differentiation, revealing a dynamic change in the overall level of O-GlcNAcylation during the process of osteogenic differentiation (Nagel et al., 2013). Moreover, Takahiro et al. Also found that the knockdown of Ets1 resulted in a significant decrease in the expression of ALP and bone sialoprotein (BSP). Prediction of glycosylation sites by Online website revealed the presence of more than 10 Ser/Thr residues in mouse Ets1 protein, which are possible modification sites for O-GlcNAc, suggesting that Ets1 may also be a potent candidate transcription factor regulated by O-GlcNAcylation (Koyama and Kamemura, 2015).

Recent studies have reported the presence of O-GlcNAc modification of several proteins in osteoblasts (Komori, 2006). Runx2 is a member of the runt-domain gene family of DNA-binding proteins (Runx1, Runx2 and Runx3). Runx2, a major transcription factor in osteoblast differentiation (Komori, 2018), is involved in the regulation of genes associated with bone matrix formation, remodeling, and mineralization, including alkaline phosphatase (ALP) and osteocalcin (OCN) (Komori, 2010). Studies have reported that post-translational modification of Runx2 by phosphorylation, ubiquitination, and acetylation modulates its activity, stability (Kim et al., 2020; Chen et al., 2021), and interaction with transcriptional co-regulators and chromatin remodeling proteins downstream of osteogenic signaling (Martin et al., 2011). Multiple studies have found that promoting O-GlcNAcylation of Runx2 enhances the transcriptional activity of Runx2 and thus participates in the regulation of osteoblast differentiation (Komori, 2006; Nagel and Ball, 2014; Sun et al., 2019). Kim et al. (2007) found elevated levels of O-GlcNAcylation during osteoblast differentiation in MC3T3-E1, which positively regulated OCN expression by increasing the transcriptional activity of Runx2. Osteoblast-specific element (OSE2), as a promoter site on OCN, is also a specific DNA binding site for Runx2 (Shui et al., 2003; Komori, 2006). Transfection of either the wild-type OCN promoter plasmid or the OSE2/32-luc plasmid PUGNAc (an inhibitor of OGA), which is located upstream of the OCN promoter and increases the copy number of OSE2, enhanced the transcriptional activity of OCN. In contrast, mutations at the OSE2 locus resulted in osteoblasts not responding to PUGNAc stimulation (Kim et al., 2007). The above results suggest that OSE2 is a key site for O-GlcNAcylation to promote OCN function. Moreover, a further increase in the expression of OCN by PUGNAc on trichostatin-induced osteoblasts was found after transfection of Runx2 plasmid and OCN promoter plasmid in COS-7 cells. Thus, elevated O-GlcNAcylation increases OCN transcription via OSE2 and Runx2 during osteoblast differentiation (Komori, 2006). In addition, BMPs can stimulate osteoblast differentiation through increased Runx2 phosphorylation and reverse transcription activation (Ge et al., 2009; Ge et al., 2012). It has been found that BMP2/7 stimulation reduced OGA activity (Nagel and Ball, 2014), suggesting that O-GlcNAcylation may be associated with osteoblast differentiation induced by BMPs.

TGF-β-activated protein kinase 1 (TAK1) is an important intracellular signaling protein kinase with serine/threonine protein kinase activity. Osteoblast function is closely related to the regulatory subunit, TAK1 binding proteins 1 (TAB1)/TAB2, associated with TAK1 signaling node2 (Nagel et al., 2013). Multiple studies have reported the presence of O-GlcNAcylation of several proteins on major components of the TAK1 signaling node (Nagel et al., 2013) including CREB-binding protein (CBP), TAB1 (Schimpl et al., 2010; Alfaro et al., 2012; Trinidad et al., 2012), TAB2 (Parker et al., 2011), TAB3 (Alfaro et al., 2012; Trinidad et al., 2012), and TAK1 (Alfaro et al., 2012). Stimulators such as IL-1β, TGFβ, and BMPs can activate TAK1 binding to the TAK1 complex (TAK1, TAB1, TAB2, and TAB3) to initiate downstream signaling cascades. The TAK1 complex has been reported to regulate Runx2 and CBP activity through O-GlcNAc modification, which in turn increases BMP2 transcription and affects osteoblast differentiation (Greenblatt et al., 2010). CBP is a histone acetyltransferase and transcriptional coactivator. In osteoblasts, CBP interacts with Runx2 and CREB to regulate their transcriptional activity, which contributes to CREB-mediated BMP2 expression, thus affecting osteoblast differentiation (Shim et al., 2012). In addition, a bioinformatics analysis proposed that redistribution of O-GlcNAcylation at different molecular weight levels could regulate the expression of osteogenic markers, thereby facilitating the transition from early to late stages of osteoblastic differentiation. Further bioinformatics analyses revealed that O-GlcNAc modification of factors such as Ctnnb1, Sp1, and Trps1 could influence the process of osteogenic differentiation, although not experimentally verified (Weng et al., 2021). Moreover, this team also proposed that the transition of osteoblasts between proliferation and differentiation requires fine regulation of calcium homeostasis (Zayzafoon, 2006). Increased or decreased cytoplasmic Ca^2+^ can inhibit the expression of osteogenic markers (Ljunggren et al., 1991; Dean et al., 2008; Noack et al., 2015; Mirsaidi et al., 2017; Pilquil et al., 2020). Intracellular Ca^2+^ concentration increased with elevated levels of O-GlcNAcylation, while changes in Ca^2+^ concentration also affected OGT expression (Weng et al., 2021), indicating that there is an interaction between intracellular O-GlcNAcylation levels and calcium signaling pathways during osteoblast differentiation.

Hyperglycemia can affect the expression of osteogenic genes and bone formation, leading to diabetic OP in a diabetic mouse model (Schwartz, 2003; Botolin and McCabe, 2006; Strotmeyer et al., 2006; Botolin and McCabe, 2007). Previous studies have reported that limiting glucose concentration promotes osteogenic differentiation of MSCs (Lo et al., 2011). It is well known that hyperglycemia promotes protein O-GlcNAcylation. Therefore, hyperglycemia-induced excessive protein O-GlcNAcylation may lead to diminished osteogenic differentiation and diabetic OP. Hanna et al. (Gu et al., 2018) demonstrated that excess O-GlcNAcylation induced by high glucose, glucosamine, N-acetylglucosamine treatment or OGT overexpression inhibited BMP2-induced osteogenic differentiation, as evidenced by the downregulation of the expression levels of ALP, COL-I, and OCN, as well as by attenuated activity of Runx2 and Osterix. These results are consistent with the reduced bone formation phenotype observed in patients with type 2 diabetes. Paradoxically, however, other studies have shown that upregulation of O-GlcNAcylation by the addition of OGA inhibitors promotes osteogenic differentiation and increases Runx2 transcriptional activity and matrix mineralization (Komori, 2006; Nagel and Ball, 2014). Contributing to these differences may be the possible different effects of metabolic treatment (high glucose concentrations) and pharmacological treatment (OGT/OGA inhibitors). Pharmacological inhibition of OGA increases O-GlcNAcylation levels by breaking the dynamic on/off cycle, whereas metabolic treatments or OGT overexpression increase O-GlcNAcylation levels by shifting the balance toward modification (Vaidyanathan and Wells, 2014). In addition, the concentration of UDP-GlcNAc, the donor of O-GlcNAcylation, may affect the selectivity of OGT substrates (Cheung et al., 2008; Cheung and Hart, 2008), suggesting that the increase in UDP-GlcNAc concentration induced by metabolic treatments may differentially regulate the O-GlcNAc modification site or the type of modified proteins in Runx2 (Yang and Qian, 2017). Thus, a moderate increase in protein O-GlcNAcylation promotes osteogenic differentiation, but an excessive increase in O-GlcNAcylation may inhibit osteogenic differentiation of cells, suggesting that the overall O-GlcNAcylation level should be maintained in an optimal range to protect normal cellular function.

Bone mesenchymal stem/stromal cells (BMSCs) have the potential for multidirectional differentiation into osteoblasts, adipocytes, chondrocytes, and myoblasts (Uzieliene et al., 2019; Huang et al., 2021). Recent studies have shown that inhibiting intracellular O-GlcNAcylation levels suppressed osteogenic differentiation of BMSCs. Specific knockdown of OGT in mouse BMSCs inhibited bone formation and reduced B Lymphopoiesis, whereas bone marrow adipocyte production was enhanced (Zhang Z. et al., 2023). The balance between osteogenic and adipogenic differentiation of BMSCs is coordinately regulated by post-translational modifications via O-GlcNAc modification by the transcription factors Runx2 and C/EBPβ. Increased O-GlcNAcylation of Runx2 is not only essential for the promotion of osteogenic differentiation but also promotes B-lymphocyte production through activation of IL-7. OGT knockdown was able to increase the transcriptional activity of C/EBPβ by inhibiting O-GlcNAcylation of C/EBPβ, which promotes adipogenic differentiation of BMSCs and increases myelopoiesis through activation of the expression of myelopoietic stem cell factor (SCF) encoded by the Kitl gene (Cordeiro Gomes et al., 2016; Asada et al., 2017; Fistonich et al., 2018; Zhang et al., 2019). In addition, elevated O-GlcNAcylation promotes osteogenic differentiation and calcification in vascular smooth muscle cells (VSMCs) (Heath et al., 2014; Zhang W. et al., 2023). STIM1 deletion in VSMCs leads to increased intracellular calcium flux, CaMKII activation and endoplasmic reticulum stress, which promotes VSMC calcification through upregulation of O-GlcNAcylation (Zhang W. et al., 2023), which may be related to the activation of Akt and Runx2 upregulation (Heath et al., 2014).

In summary, the existing evidence suggests that multiple proteins critical for the regulation of bone formation and bone remodeling are regulated by O-GlcNAcylation. Therefore, pharmacological inhibition of OGA/OGT to reduce protein O-GlcNAcylation could be used as an effective potential strategy for bone metabolic diseases. In addition, further studies are needed to investigate the potential mechanism of protein O-GlcNAcylation in diabetic OP in OGT and OGA conditional knockout animal models.

### 3.3 O-GlcNAcylation and chondrocytes

In the skeletal system, chondrocytes are primarily responsible for maintaining and repairing cartilage tissue. Degeneration of cartilage and disruption of homeostasis are important pathologic changes in several bone diseases, including OA (LuValle and Beier, 2000; De Luca, 2006). The chondrogenic differentiation cascade of events includes cell proliferation, extracellular matrix synthesis, cell hypertrophy, matrix mineralization, vascular invasion, and ultimately apoptosis, which results in the remodeling of cartilage into bone tissue (Kronenberg, 2003). Studies have shown that insulin can induce chondrocyte hypertrophy and differentiation in a dose-dependent manner (Mueller et al., 2013). However, the underlying mechanisms are not fully understood. Jessic et al. (Andrés-Bergós et al., 2012) demonstrated for the first time that insulin-induced chondrocyte differentiation (chondrocyte differentiation) is mediated through the protein O-GlcNAcylation. The team found that insulin treatment significantly upregulated the expression of chondrocyte differentiation-related markers such as parathyroid hormone receptor type 1 (PTH1R), Runx2 and collagen X (COL-X). Inhibition of the protein O-GlcNAcylation impeded insulin-induced elevated expression of markers of chondrogenic differentiation. Further studies showed that thiamet-G-induced elevation of O-GlcNAcylation in the absence of insulin was able to significantly promote chondrogenic differentiation. Another important hallmark of chondrocyte differentiation is the enzymatic remodeling of the extracellular matrix. The matrix metalloproteinase family (MMPs) is a family of solubilizing enzymes that play an irreplaceable role in the remodeling and degradation of the extracellular matrix (91, 92). Increased O-GlcNAcylation was found to upregulate MMP-9 and MMP-2 activity (Andrés-Bergós et al., 2012). Previous studies have demonstrated that increased MMP-9 and MMP-2 activity correlates with chondrocyte differentiation (Challa et al., 2010). Thus, increased protein O-GlcNAcylation may promote chondrocyte differentiation by upregulating MMP-9 and MMP-2 activity, although further experimental validation is needed.

Mature intervertebral disc cells are chondrocyte-like cells. Nikolaou et al. (2017) showed that enhanced expression of the proteins O-GlcNAcylation and OGA/OGT was observed in tissues of human intervertebral disc degeneration (IDD), suggesting that an increase in O-GlcNAcylation is closely related to chondrocyte apoptosis and disc degeneration. Unfortunately, the study lacked normal intervertebral disc tissue samples as a control. However, Luo et al. (2022) found that OGT expression and O-GlcNAcylation were elevated in degenerated nucleus pulposus tissue and nutritionally deficient nucleus pulposus cells compared to nucleus pulposus cells in healthy disc tissue. Furthermore, inhibition of O-GlcNAcylation promoted the induction of apoptosis and senescence in human nucleus pulposus cells by nutrient deficiency. Under nutrient-deficient conditions, FAM134B was able to directly interact with OGT to undergo O-GlcNAcylation, which enhanced the stability of FAM134B protein by inhibiting ubiquitinated degradation to promote FAM134B-mediated autophagy in the endoplasmic reticulum and inhibit apoptosis and senescence in myeloid cells and ultimately delayed the progression of IDD (Luo et al., 2022). It has been proposed that the intervertebral disc is an avascular tissue with a hypoglycemic microenvironment (Boskey, 2008). The cartilaginous endplate is a transparent cartilaginous layer that separates the intervertebral disc from the vertebral body. The glucose concentration was significantly higher on the outside of the cartilage endplate than on the inside. Its internal glucose concentration was as low as 1 mM (Shirazi-Adl et al., 2010). Chondrogenic endplate stem cells (CESCs) derived from human cartilage endplates are a type of stem cells that have greater chondrogenic and osteogenic differentiation potential than BMSCs. Some scholars have induced chondrogenic and osteogenic differentiation of CESCs isolated from degenerated/healthy cartilage endplates under low glucose (1 mM), normal glucose (5 mM) and high glucose (25 mM) concentration culture conditions, respectively (As shown in Figure 2) (Sun et al., 2019). The results demonstrated that glucose in the microenvironment is essential for regulating CESC chondrogenesis and osteogenic differentiation, which may be closely related to O-GlcNAcylation. High glucose promotes CESCs toward osteogenic differentiation and decreases chondrogenic differentiation capacity by increasing O-GlcNAcylation, whereas low glucose promotes CESCs toward chondrogenic differentiation. Sox9 is a key transcription factor that promotes cell survival and transcriptionally activates several cartilage-specific markers. O-GlcNAcylation of Sox9 inhibits its activity and decreases the expression of Sox9 downstream factor COL-II (Sun et al., 2019). Further, Runx2 activity was also positively correlated with O-GlcNAcylation levels in CESCs (Komori, 2006; Nagel and Ball, 2014). Thus, O-GlcNAcylation regulation by Sox9 and Runx2 provides new drug targets for degenerative disc disease. Moreover, DNA methylation, a newly discovered epigenetic modification, has been found to play an important regulatory role in chondrocyte differentiation. TET1 (tet methylcytosine dioxygenase 1), an enzyme that induces DNA demethylation, is considered a key epigenetic factor in the hypertrophic differentiation of chondrocytes, which is highly expressed during chondrogenesis (Vágó et al., 2021). OGT is able to regulate the biological activity of TET enzymes. It was shown that OGT interacts with TET1 to increase the stability and activity of TET1 (Hrit et al., 2018). It is hypothesized that OGT may promote O-GlcNAcylation of TET1 to activate DNA demethylation, thereby regulating chondrogenesis and chondrogenic differentiation.

In summary, OGT-mediated O-GlcNAcylation is closely associated with the stability, activity, and function of multiple markers related to chondrogenesis and chondrocyte differentiation. Regulating OGT/O-GlcNAcylation may be a potential strategy to maintain cartilage homeostasis. In addition, O-GlcNAcylation interacts extensively with other post-translational modifications such as phosphorylation and ubiquitination. This complex regulatory relationship also requires further elucidation to help better understand the regulation of cartilage homeostasis.

## 4 O-GlcNAcylation and bone-related diseases

### 4.1 O-GlcNAcylation and osteoporosis

OP is a bone metabolic disease characterized by low bone density and bone mass, destruction of bone microstructure leading to increased bone fragility and increased risk of fracture. The uncoupling of bone resorption and bone formation is the most important cause of the development of this disease. Several studies have shown that O-GlcNAc affects osteoblast and osteoclast differentiation by modifying marker proteins during bone metabolism to influence their stability. Kim et al. (2021) found that local injection of OSMI-1, an inhibitor of OGT, effectively inhibited lipopolysaccharide (LPS)-induced formation of TRAP-positive osteoclasts and reduced the number of TRAP-specific mature osteoclasts, which led to a decrease in the cranial bone mineral density in mice. It is well recognized that the progression of many multiple cancers into bone metastases can lead to bone destruction and even pathologic fractures (Wu et al., 2020). Notably, studies have reported that OGT is not only highly expressed in many cancers, but there is a positive correlation between OGT and cancer metastatic progression (Esposito et al., 2021). To further understand the relationship between O-GlcNAcylation and bone metastasis, some scholars compared the efficacy of OGT-targeted inhibition with the bone-targeted therapeutic drug zoledronic acid. The results found that OGT inhibition and bone-targeted therapy had a synergistic effect on osteoclastogenesis (Kim et al., 2021). Another study found that inhibiting osteoclast differentiation and ameliorating inflammation-induced bone loss in a mouse model of arthritis by modulating O-GlcNAcylation (Li et al., 2022). Thus, targeted modulation of O-GlcNAcylation levels can not only inhibit arthritis-associated bone resorption under inflammatory conditions but also alleviate OP-associated bone resorption under non-inflammatory conditions. In addition to inhibiting osteoclast activity, Zhang Z. et al. (2023) found that OGT knockout mice had reduced bone volume and number of osteoblasts, decreased bone density, and defects in bone and tooth development, demonstrating that O-GlcNAcylation may also be involved in regulating bone formation. Furthermore, Min et al. (Cai et al., 2023) determined that the key protein SIRT1, a class of nicotinamide adenine dinucleotide (NAD+) dependent deacetylases, may be involved in the regulation of bone remodeling by mass spectrometry PPI network analysis and hub gene screening comparisons of fresh blood samples from postmenopausal women with OP and a healthy population. Mass spectrometry and PyMol structure analysis revealed that SIRT1 asparagine at position 346 underwent O-GlcNAcylation. Under stress stimulation, the O-GlcNAcylation level of SIRT1 was elevated and bound to NAD + through Pi-Pi interaction, which enhanced the SIRT1 deacetylase activity and deacetylated RANKL protein, thus inhibiting RANKL-mediated transcriptional activation to suppress osteoclast differentiation without affecting their survival and regulating the balance between osteoblast survival and apoptosis. This suggests that the O-GlcNAcylation of SIRT1 is closely related to the regulation of bone metabolism, which may provide a theoretical basis for the prevention and treatment of OP.

In summary, O-GlcNAc may affect bone metabolism by modifying multiple protein markers. Therefore, modulation of O-GlcNAc levels is expected to be a potential therapeutic target for OP.

### 4.2 O-GlcNAcylation and arthritis

Previous studies have proposed that dysregulation of O-GlcNAcylation affects inflammatory signaling pathways such as NF-κB, MAPK, and PI3K/AKT, which can lead to a variety of inflammatory diseases (Li et al., 2019). Arthritis is a disease primarily associated with autoimmune reactions, infections, metabolic disorders and trauma, such as osteoarthritis (OA) and rheumatoid arthritis (RA). It has been shown that O-GlcNAcylation is closely related to the pathogenesis of OA and RA mainly. OA is a chronic degenerative joint disease characterized by cartilage degeneration, synovial inflammation and subchondral bone remodeling. Pathologic changes in articular cartilage are recognized as one of the key drivers of OA. Impairment of chondrocyte function disrupts the metabolic balance between extracellular matrix synthesis and catabolism, leading to articular cartilage degeneration (Liu et al., 2020; Lubbers et al., 2020). It was shown that increased O-GlcNAcylation induced a significant expansion of the height of the growth plate and the height of the hypertrophic zone in neonatal mice and promoted hypertrophic differentiation of chondrocytes (Andrés-Bergós et al., 2012). A significant increase in O-GlcNAcylation was also found in degenerated articular cartilage in clinical samples of OA and animal models of OA (Largo et al., 2012; Tardio et al., 2014). In addition, IL-1β, a key catabolic cytokine in OA, was able to induce further accumulation of O-GlcNAc in chondrocytes from OA patients, suggesting that the inflammatory environment may contribute to the increased O-GlcNAcylation of cartilage in OA, which may exacerbate cartilage damage (Tardio et al., 2014). In addition to cartilage degeneration, the role of synovial inflammation in the pathologic changes of OA is equally noteworthy, as it is closely associated with painful symptoms and has value in determining prognosis (Wang et al., 2016; Mathiessen and Conaghan, 2017). Although glucosamine (GlcN) inhibits inflammatory factor production in synoviocytes and thus exhibits a protective effect against OA (Hua et al., 2007; Kapoor et al., 2012), the underlying anti-inflammatory mechanism of GlcN remains unclear. Since GlcN can act as an inducer of HBP, it has been speculated that GlcN has the potential to exert an anti-inflammatory effect by regulating cellular function through O-GlcNAcylation of target proteins (Hwang et al., 2013; Suh et al., 2014). Using DNA microarray analysis, a team of researchers found that more than half of the GlcN-regulated genes were mediated by O-GlcNAcylation in the IL-1β-stimulated human synovial cell line MH7A. It has also been found that GlcN can inhibit pro-inflammatory cytokine expression, while OGT inhibitors can partially restore GlcN-regulated pro-inflammatory cytokine expression (Someya et al., 2016). Thus, O-GlcNAcylation is closely related to the process of anti-inflammatory regulation of OA synoviocytes by GlcN.

RA is a common chronic and systemic autoimmune disease characterized by aggressive inflammation, overgrowth of synovial tissue, and progressive joint erosion, ultimately leading to loss of joint function and deformity (Weyand and Goronzy, 2021). It has been shown that O-GlcNAcylation can affect macrophage function to regulate the immune response process in the organism (Jitschin et al., 2019; Yang et al., 2020). Accumulating evidence shows that O-GlcNAcylation is highly expressed in fibroblast-like cells and synovial tissues of RA patients compared to normal tissues. The inflammatory milieu in RA promotes O-GlcNAcylation levels (Li et al., 2022; Umar et al., 2022). Inhibition of O-GlcNAcylation of TAB1 in fibroblast-like cells from RA patients reduces TAK1 autophosphorylation and suppresses TAK1 activation (Umar et al., 2022). Phosphorylation of TAK1 is an important determinant of downstream signaling in human RASFs. Induction of TAK1 activation leads to activation of the downstream pathways MAPK and IKK thereby promoting the release of inflammatory mediators and tissue destruction in RA (Ajibade et al., 2013). Kim et al. (2015) found that increased O-GlcNAcylation promoted TNF-α-stimulated proliferation of fibroblast-like synoviocytes and expression of RA-associated proinflammatory cytokines, and exacerbated synovial proliferation and bone destruction in RA. O-GlcNAcylation of the NF-κB subunit p65 can increase the expression levels of downstream genes by promoting nuclear translocation of p65, DNA binding of proteins, and enhanced transcriptional activity, thereby enhancing the TNF-α-induced inflammatory process. In addition, Th1 and Th17 cells play a key role in the pathogenesis of RA. O-GlcNAcylation of p65 may exacerbate the severity of arthritis by contributing to the transformation of T cell populations into Th1 and Th17 cells (Lee et al., 2013; Kim et al., 2015).

Thus, O-GlcNAcylation is involved in different pathological processes in arthritis, such as chondrocyte hypertrophy, synovial proliferation and inflammation. The modulation of O-GlcNAcylation may be a potential therapeutic strategy for arthritis. However, the potential regulatory mechanism of O-GlcNAcylation in arthritis needs to be further investigated.

### 4.3 O-GlcNAcylation and osteosarcoma

Osteosarcoma is one of the most common primary malignant bone tumors in adolescents with a 5-year survival rate of less than 20%. Currently, the main treatment strategy for osteosarcoma is surgery combined with chemotherapy (Isakoff et al., 2015). Some studies have revealed the presence of high expression of OGT and O-GlcNAcylation in different solid tumor tissues. O-GlcNAcylation can affect tumor glucose metabolism, metastasis, proliferation, vascular invasion and drug resistance by modifying tumor-associated proteins (Cheng et al., 2016; He et al., 2023). Recently, a clinical study examined O-GlcNAcylation, OGT, and OGA expression in bone specimens derived from 109 patients diagnosed with osteosarcoma. The results showed that high OGA expression was significantly associated with longer overall survival and metastasis-free period in patients with stage IIB, and positively correlated with good response to chemotherapeutic agents (i.e., tumor tissue necrosis percentage ≥90). Further multivariate analysis indicated that OGA expression was an independent predictor of good prognosis for osteosarcoma (Sombutthaweesri et al., 2022). In addition, cancer cells are able to promote signaling pathways associated with tumorigenesis by increasing the uptake of glucose and glutamine, which can upregulate the flux of the HBP pathway and increase O-GlcNAcylation levels (Lee J. B. et al., 2021). It has been shown that HBP pathway dysregulation is significantly associated with the prognosis of osteosarcoma patients. Four HBP-related genes (GFPT, GNPNAT, PGM3, and UAP1) can be used to guide immunologic and targeted therapies for osteosarcoma, with significant predictive value for osteosarcoma prognosis (Su et al., 2022).

ROCK2 can modulate the level of target protein phosphorylation/activity to regulate downstream gene expression. ROCK2 has been shown to promote cancer cell proliferation, which is closely associated with cancer progression and poor prognosis (Calò et al., 2019). Deng et al. (2020) showed that ROCK2 could promote the proliferation of osteosarcoma cells. Overexpression of ROCK2 promotes OGT protein stability by inhibiting OGT degradation and ubiquitination through the ubiquitin-proteasome system, which ultimately promotes osteosarcoma cell proliferation and tumor growth and increases resistance to TRAIL (Deng et al., 2020). In addition, Sun et al. (2022) showed that lncEBLN3P was able to enhance OGT expression by targeting miR-200a-3p, which ultimately promotes metastasis and drug resistance in osteosarcoma. Unfortunately, the studies related to O-GlcNAcylation and osteosarcoma have rarely been validated by animal experiments.

In summary, the expression level of O-GlcNAcylation was significantly elevated in patients with osteosarcoma. O-GlcNAcylation accelerates tumor progression by regulating tumor cell proliferation, differentiation, and migration and is strongly associated with adverse clinical outcomes. Therefore, the development of O-GlcNAcylation-related specific inhibitors provides a new therapeutic direction for osteosarcoma treatment. Although O-GlcNAcylation has shown strong therapeutic potential in the treatment of osteosarcoma, the potential regulatory mechanisms of O-GlcNAcylation in osteosarcoma remain unclear. More studies are still needed to further investigate the role of O-GlcNAcylation in osteosarcoma. In addition, the efficacy and safety of O-GlcNAcylation inhibitors *in vivo* need to be validated by extensive preclinical and clinical trials.

## 5 Conclusions and perspectives

In summary, O-GlcNAcylation, as a key protein post-translational modification, plays a crucial role in bone metabolism, which affects the occurrence and prognosis of OP, OA, RA, and other diseases by participating in processes such as the formation and differentiation of bone-associated cells, including osteoblasts, osteoclasts, and chondrocytes (as shown in Table 1). The underlying complex molecular mechanism involves O-GlcNAc modification of target proteins, which impacts their activity and stability and regulates multiple signaling pathways, which in turn affects osteoblast differentiation and bone matrix mineralization, as well as osteoclast formation and activity, ultimately regulating bone metabolism. In addition, O-GlcNAcylation is involved in the regulation of multiple signaling pathways, including the RANK and NF-κB signaling pathways, which play a key role in bone metabolism by regulating the activity of bone metabolism-associated transcription factors (e.g., Runx2). Moreover, the imbalance of OGT (O-GlcNAc transferase) and/or OGA (O-GlcNAcase) also leads to changes in O-GlcNAcylation levels, which in turn affects the activity of target proteins and bone metabolic processes. Although only a small number of proteins have been found to affect bone metabolic homeostasis via O-GlcNAcylation, further studies by glycosylation proteomics technology are expected to identify more novel target proteins. Targeted therapies against O-GlcNAcylation or OGT/OGA can provide new directions in the diagnosis and treatment of bone-related diseases. The interaction between O-GlcNAcylation and other post-translational modifications, such as phosphorylation and ubiquitination, suggests the complexity of the regulation of bone metabolism. Exploring the interplay between these modifications may also provide additional avenues for therapeutic intervention. A multidisciplinary approach combining cell biology, molecular biology and clinical studies is needed in the future to fully explore the therapeutic potential of O-GlcNAcylation in bone health and disease management. In addition, future studies could delve deeper into the specific role of O-GlcNAcylation in bone biology, identify additional substrates, and establish the clinical relevance and efficacy of modulating this modification in the management of skeletal disease.

**TABLE 1:** O-GlcNAcylation regulates bone metabolism.

Authors	Cell type	Protein modified by O-GlcNAcylation	O-GlcNAcylation level	Functions
Li et al. (2022)	Mouse bone marrow-derived osteoclasts, RAW264.7 cells	NUP153↑	Promoting	Promoting osteoclast differentiation
Kim et al. (2021)	Mouse bone marrow-derived osteoclasts	NF-kB p65/NFATc1↑	Promoting	Promoting osteoclast differentiation
Takeuchi et al. (2020)	Mouse primary bone marrow cells, RAW264 cells and Human PBMCs	vimentin ↑	Promoting	Inhibiting osteoclast differentiation
Takeuchi et al. (2017)	RAW264 cells	—	Promoting	Inhibiting osteoclast differentiation
Takeuchi et al. (2016)	RAW264 cells, human PBMCs	phosphorylation of NF-κB p65↓	Promoting	Inhibiting osteoclast differentiation
Koyama and Kamemura (2015)	RAW264 cells	—	Promoting	osteoclast differentiation is unaffected
Koyama and Kamemura (2015)	MC3T3-E1 cells	Ets1, Runx2↑	Promoting	Promoting osteoblast differentiation
Gu et al. (2018)	C2C12 cell, hPDL cell	Runx2↑	Promoting excessive O-GlcNAcylation	Inhibiting osteoblast differentiation
Zhang et al. (2023a)	BMSCs	Runx2↑	Promoting	Promoting osteoblast differentiation
Nagel et al. (2013)	Primary human osteoblasts (NHOst), MC3T3-E1	TAB1, TAB2, CBP↑	Promoting	Promoting osteoblast differentiation
Weng et al. (2021)	MC3T3-E1	—	Promoting	Promoting osteoblast differentiation
Nagel and Ball (2014)	BMSCs, MC3T3-E1	Runx2↑	Promoting	Promoting osteoblast differentiation
Zhang et al. (2023b)	VSMCs	—	Promoting	Promoting VSMC osteogenic differentiation and calcification
Heath et al. (2014)	VSMCs	phosphorylation of AKT↑	Promoting	Promoting VSMC osteogenic differentiation and calcification
Kim et al. (2007)	MC3T3-E1	Runx2↑	Promoting	Promoting osteoblast differentiation
Cai et al. (2023)	hFOB1.19 cells	the deacetylation activity of SIRT1↑	Promoting	Promoting osteoblast differentiation
Sun et al. (2019)	Cartilage endplate stem cells	Sox9↑, Runx2↑	Promoting	Inhibiting chondrogenesis and Promoting osteogenesis
Vágó et al. (2021)	Mouse chondrocytes	TET1↑	Promoting	Promoting chondrogenic differentiation
Andrés-Bergós et al. (2012)	ATDC5, mouse chondrocytes	—	Promoting	Promoting chondrocyte differentiation
Nikolaou et al. (2017)	Intervertebral disc cells	—	Promoting	Promoting chondrocyte apoptosis
Luo et al. (2022)	Human nucleus pulposus cells	FAM134B↑	Promoting	Inhibiting chondrocyte apoptosis
Tardio et al. (2014)	Human OA chondrocytes, human healthy chondrocytes	—	Promoting	Promoting chondrocyte hypertrophic differentiation
